# Supplementary material for: The CFTR Amplifier Nesolicaftor Rescues TGF-β1 Inhibition of Modulator-Corrected F508del CFTR Function
Source: Int J Mol Sci. 2022 Sep 19;23(18):10956. doi: 10.3390/ijms231810956 (PMC9504033; doi:10.3390/ijms231810956)
Supplement: Supplementary file 1 [file ijms-23-10956-s001.zip › ijms-1915410-supplementary.pdf]

Supplementary Materials

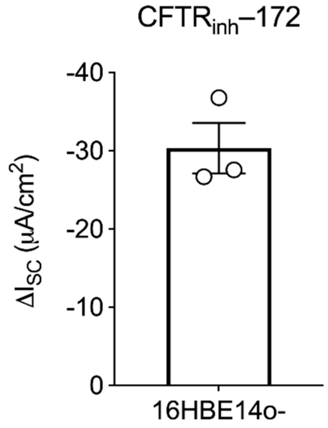

Supplementary Figure S1. CFTR-dependent short-circuit currents (I_{SC}) measured in Ussing chambers from confluent monolayers of the wild-type 16HBE14o- cell line.

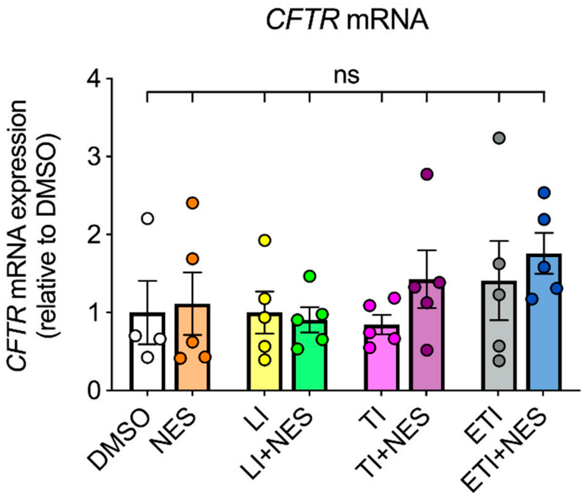

Supplementary Figure S2. Effects of nesolicaftor (NES) on *CFTR* mRNA expression levels in modulator-corrected F508del-CFTR 16HBEge cells *in vitro*. Confluent monolayers of F508del-CFTR 16HBEge cells were treated with DMSO, NES (10 μM), lumacaftor (5 μM)/ivacaftor (1 μM) (LI), LI + NES, tezacaftor (5 μM)/ivacaftor (1 μM) (TI), TI + NES, elexacaftor (1 μM)/tezacaftor (5 μM)/ivacaftor (1 μM) (ETI), or ETI + NES both apically and basolaterally for 24 h. Nesolicaftor does not significantly change expression levels of *CFTR* mRNA under any treatment condition. *Statistics:* Kruskal-Wallis test after assessing normality with Shapiro-Wilk. ns = not significant.

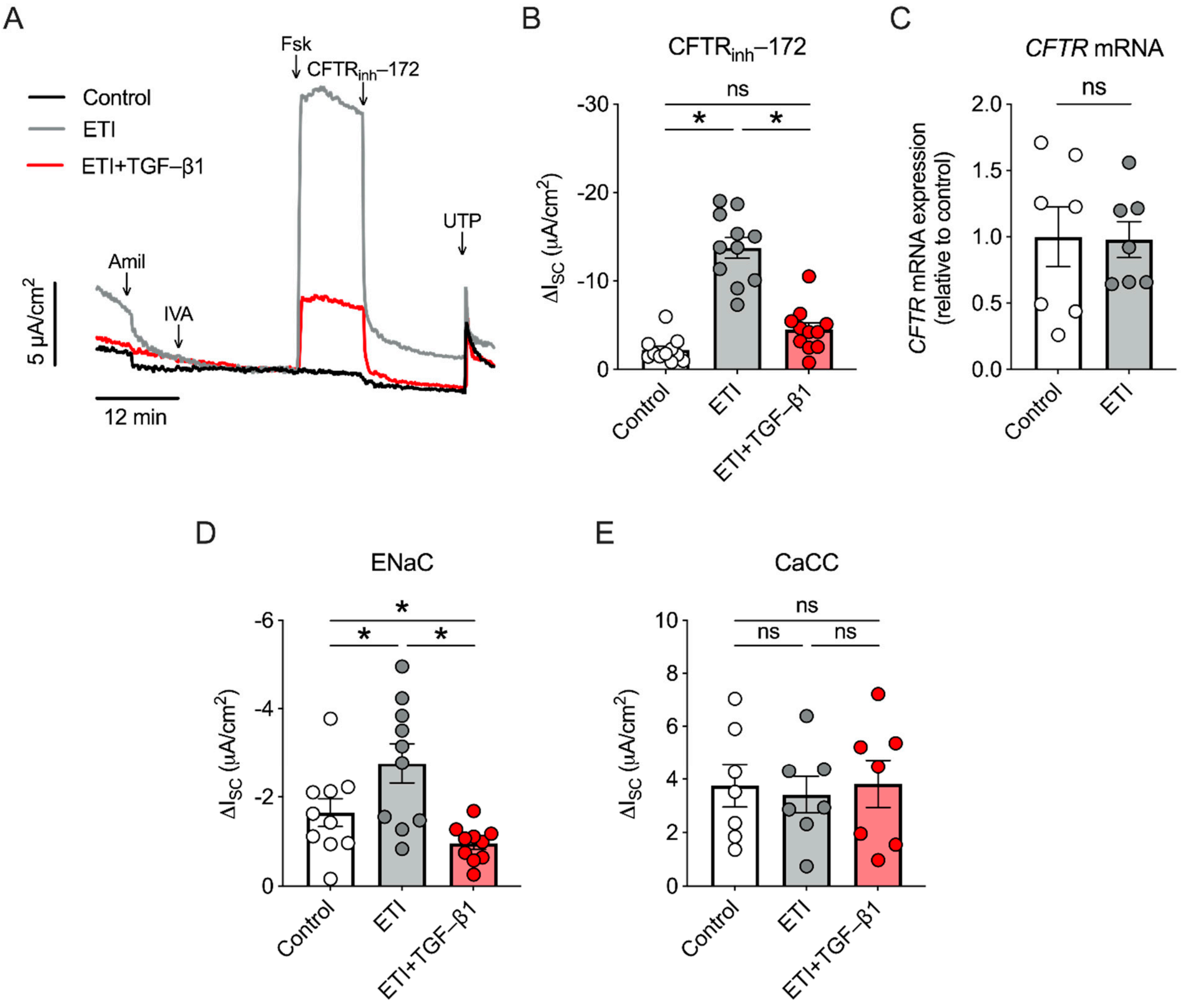

Supplementary Figure S3. Effects of ETI and TGF-β1 on ion channel function in primary CFBE cells *in vitro*. (**A**) Fully differentiated ALI cultures of primary human CFBE cells homozygous for F508del were treated with DMSO (control), elexacaftor (1 µM)/tezacaftor (5 µM)/ivacaftor (1 µM) (ETI), or ETI + TGF-β1 (5 ng/mL) for 24 h and CFTR-dependent I_{SC} was measured in Ussing chambers. (**B**) ETI causes a significant increase in F508del-CFTR function after 24 h, an effect inhibited by TGF-β1. n = 12, 5 CF lungs. (**C**) ETI does not change expression levels of *CFTR* mRNA after 24 h. n = 7, 4 CF lungs. (**D**) ETI causes a significant increase in ENaC conductance after 24 h. TGF-β1 causes a significant decrease in ENaC function. n = 10, 5 CF lungs. (**E**) ETI and TGF-β1 do not change CaCC activity after 24 h. n = 7, 5 CF lungs. *Statistics:* * $p < 0.05$, Friedman test (A), Student's t-test (B), or one-way ANOVA followed by Holm-Sidak (D,E) after assessing normality with Shapiro-Wilk. ns = not significant.

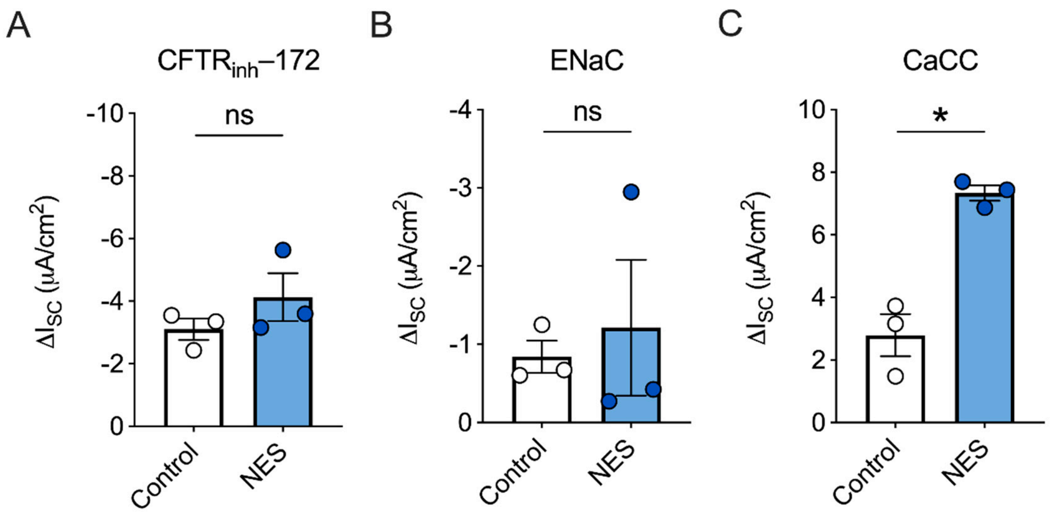

Supplementary Figure S4. Effects of nesolicaftor (NES) on ion channel function in primary CFBE cells *in vitro*. (**A**) Fully differentiated ALI cultures of primary human CFBE cells homozygous for F508del were treated with DMSO (control) or NES (10 µM) for 24 h. (**A,B**) Nesolicaftor did not cause a significant increase in CFTR (**A**) or ENaC (**B**) conductance after 24 h. n = 3 CF lungs. (**C**) Nesolicaftor significantly increases CaCC activity in CFBE cells after 24 h. n = 3 CF lungs. *Statistics:* * $p < 0.05$, Student's t-test after assessing normality with Shapiro-Wilk. ns = not significant.

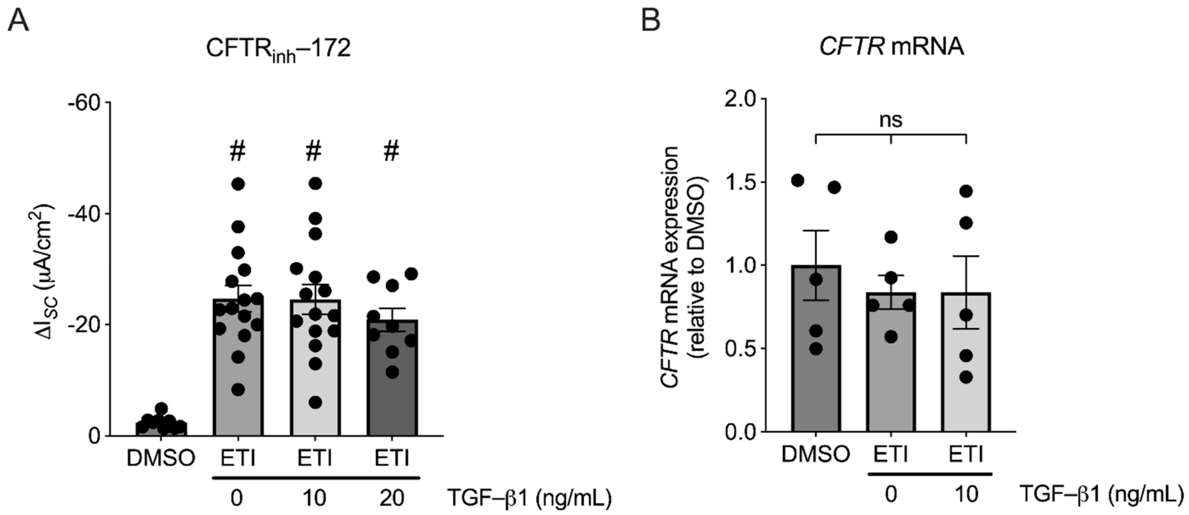

Supplementary Figure S5. Effects of TGF-β1 on ETI-corrected F508del-CFTR function and *CFTR* mRNA expression in F508del-CFTR 16HBEge cells *in vitro*. (**A,B**) Confluent monolayers of F508del-CFTR 16HBEge cells were treated with DMSO or elexacaftor (1 μM)/tezacaftor (5 μM)/ivacaftor (1 μM) (ETI) with indicated concentrations of TGF-β1 both apically and basolaterally for 24 h. TGF-β1 does not significantly change CFTR conductance (**A**) or expression levels of *CFTR* mRNA (**B**). *Statistics:* # $p < 0.05$ compared to DMSO control, one-way ANOVA followed by Holm-Sidak after assessing normality with Shapiro-Wilk. ns = not significant.

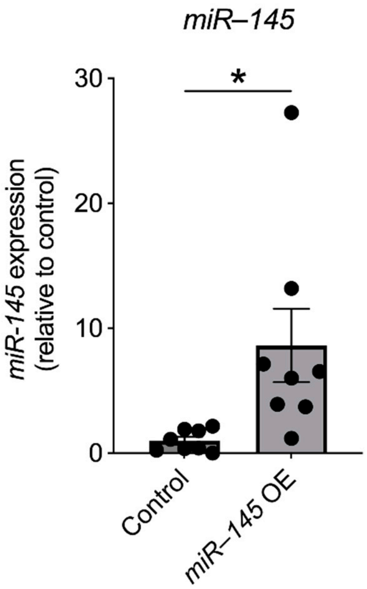

Supplementary Figure S6. Levels of *miR-145* expression in primary CFBE cells transduced with *miR-145* lentivirus compared to control. *Statistics:* * $p < 0.05$, Mann-Whitney test after assessing normality with Shapiro-Wilk.
